# *Tomo Live*: an on-the-fly reconstruction pipeline to judge data quality for cryo-electron tomography workflows

**DOI:** 10.1107/S2059798324001840

**Published:** 2024-03-21

**Authors:** Maxime Comet, Patricia M. Dijkman, Reint Boer Iwema, Tilman Franke, Simonas Masiulis, Ruud Schampers, Oliver Raschdorf, Fanis Grollios, Edward E. Pryor, Ieva Drulyte

**Affiliations:** a Materials and Structural Analysis, Thermo Fisher Scientific, Achtseweg Noord 5, 5651 GG Eindhoven, The Netherlands; University of Exeter, United Kingdom

**Keywords:** electron tomography, real-time processing, cryo-ET, *Tomo Live*

## Abstract

Data acquisition and processing for cryo-electron tomography can be a significant bottleneck for users. To simplify and streamline the cryo-ET workflow, *Tomo Live*, an on-the-fly solution that automates the alignment and reconstruction of tilt-series data, enabling real-time data-quality assessment, has been developed. Through the integration of *Tomo Live* into the data-acquisition workflow for cryo-ET, motion correction is performed directly after each of the acquired tilt angles. Immediately after the tilt-series acquisition has completed, an unattended tilt-series alignment and reconstruction into a 3D volume is performed. The results are displayed in real time in a dedicated remote web platform that runs on the microscope hardware. Through this web platform, users can review the acquired data (aligned stack and 3D volume) and several quality metrics that are obtained during the alignment and reconstruction process. These quality metrics can be used for fast feedback for subsequent acquisitions to save time. Parameters such as Alignment Accuracy, Deleted Tilts and Tilt Axis Correction Angle are visualized as graphs and can be used as filters to export only the best tomograms (raw data, reconstruction and intermediate data) for further processing. Here, the *Tomo Live* algorithms and workflow are described and representative results on several biological samples are presented. The *Tomo Live* workflow is accessible to both expert and non-expert users, making it a valuable tool for the continued advancement of structural biology, cell biology and histology.

## Abstract 2

To simplify the cryo-electron tomography workflow and make this technique more accessible to all researchers, the *Tomo Live* software has been developed, which performs on-the-fly reconstruction of tilt series, enabling real-time data-quality monitoring, curation and export.

## 1. Introduction

**Figure 1:**
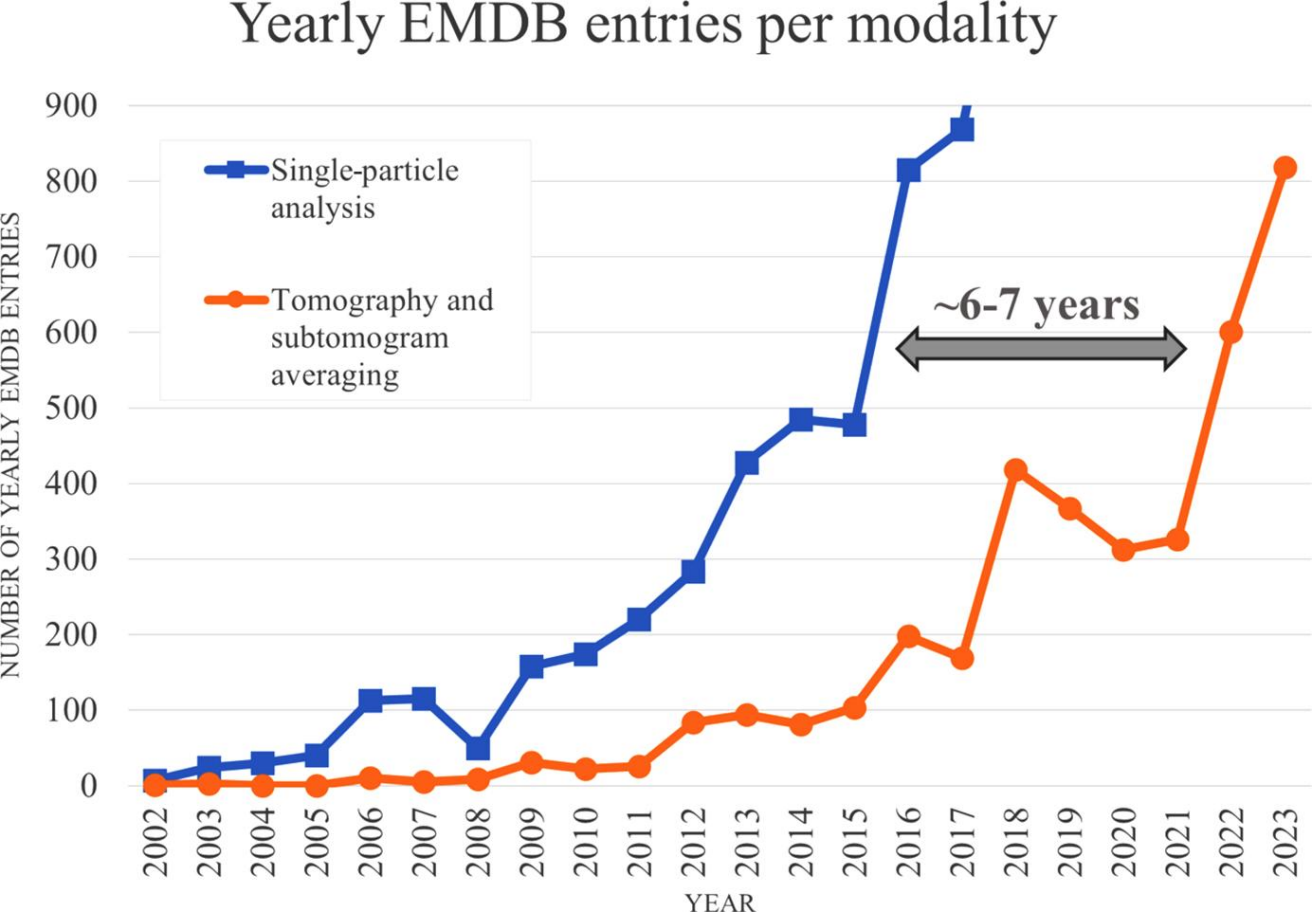
Number of EMDB depositions per year (last updated 31 October 2023). Source: https://www.ebi.ac.uk/emdb/statistics/emdb_modality_by_year.

Cryo-electron tomography (cryo-ET) is growing in popularity due to its ability to provide an overview of the cellular landscape at the nanometre scale (Hylton & Swulius, 2021 ▸; Turk & Baumeister, 2020 ▸). This is driven by parallel innovations in sample preparation, imaging and data-processing technology (Berger *et al.*, 2023 ▸; Rigort *et al.*, 2012 ▸; Tacke *et al.*, 2021 ▸; Bharat & Scheres, 2016 ▸; Scaramuzza & Castaño-Díez, 2021 ▸) and is shown by the acceleration of yearly tomography and subtomogram averaging depositions in the Electron Microscopy Data Bank (EMDB; Fig. 1 ▸). This growth mirrors the trajectory seen for cryo-electron microscopy single-particle analysis (cryo-EM SPA, or just SPA) approximately 6–7 years ago. Nonetheless, the throughput and ease of use of the data-acquisition and processing workflow for cryo-ET lag behind those of SPA.

Many of the improvements in SPA data acquisition in recent years are due to the incorporation of real-time data processing (Bepler *et al.*, 2022 ▸). The benefits of real-time data processing include efficient screening, failing early on non-optimal samples and enabling on-the-fly optimization and adjustment of data-acquisition parameters. Data-acquisition programs such as *Smart EPU*, *SmartScope* (Bouvette *et al.*, 2022 ▸) and *Smart Leginon* (Cheng *et al.*, 2023 ▸) have incorporated various implementations of real-time data processing, while programs such as *CryoFLARE* (Schenk *et al.*, 2020 ▸), *cryoSparc Live* (Punjani *et al.*, 2017 ▸), *RELION* 4.0 (Kimanius *et al.*, 2021 ▸), *Warp* (Tegunov & Cramer, 2019 ▸) and *SIMPLE* 3.0 (Caesar *et al.*, 2020 ▸) enable processing of data in real time.

Analogously to SPA, the cryo-ET workflow is undergoing continuous development, from sample preparation to data processing and reconstruction, to improve throughput and ease of use. To evaluate the quality of tomograms acquired in a cryo-ET experiment prior to subtomogram averaging, several downstream processing steps need to be performed, including motion correction of individual tilt movies, defocus estimation and tilt-series alignment (Pyle & Zanetti, 2021 ▸). These steps are generally performed using software after the acquisition such as *MotionCor*2 (Zheng *et al.*, 2017 ▸), *CTFFIND* (Rohou & Grigorieff, 2015 ▸), *IMOD* (Mastronarde & Held, 2017 ▸), *EMAN*2 (Tang *et al.*, 2007 ▸) and *Warp* (Tegunov & Cramer, 2019 ▸), among many others. For non-expert users, these steps can be challenging, and even for experts they can be quite time-consuming. The recent introduction of *AreTomo* (Zheng *et al.*, 2022 ▸), *TOMOMAN* (Khavnekar *et al.*, 2023 ▸), *TomoBEAR* (Balyschew *et al.*, 2023 ▸) and *nextPYP* (Liu *et al.*, 2023 ▸) simplifies the cryo-ET workflow by automating the processing pipeline to generate reconstructed tomograms which can be used in subsequent steps within the same or an outside program, such as *Dynamo* (Scaramuzza & Castaño-Díez, 2021 ▸), for subtomogram averaging. Nonetheless, it is worth noting that these tools do not directly interface with the microscope hardware, limiting the possibilities for integration with data-acquisition software to facilitate potential future automation and feedback loops.

**Figure 2:**
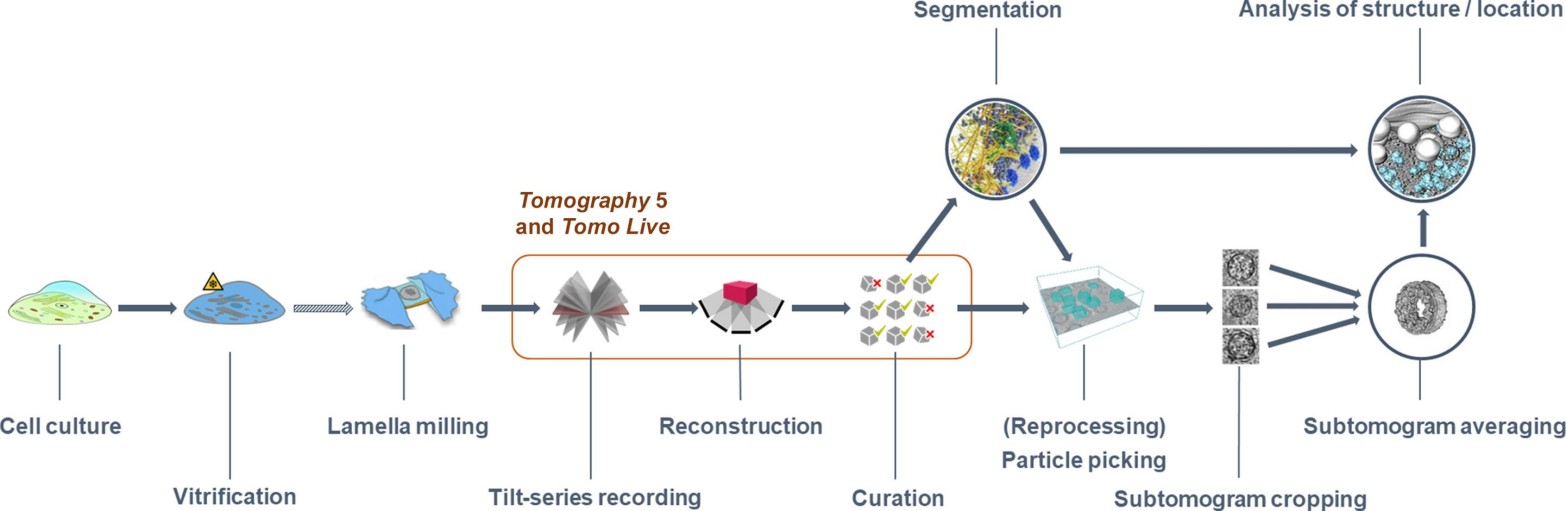
Cryo-electron tomography workflow demonstrating where the *Tomography* 5 and *Tomo Live* software fit.

To provide a fully integrated solution for real-time data acquisition and processing of cryo-ET data, we present *Tomo Live*, a software package that enables on-the-fly quality monitoring of data acquired with the *Tomography* 5 software and runs on most existing microscope hardware configurations. *Tomo Live* performs motion correction of dose-fractionated movies, fiducial-free tilt-series alignment and 3D reconstruction without user intervention during tilt-series recording (Fig. 2 ▸). These reconstructed tomograms allow impromptu data-quality evaluation, the identification of tilt series that are suitable for processing and the export of selected tilt series and reconstructed tomograms within a web-based interface for further processing, archiving and sharing.

Here, we describe the *Tomo Live* processing workflow as well as recent *Tomography* 5 software improvements aimed at ease of use and increased throughput. As illustrative examples, we show tomograms of several biological samples that were produced by *Tomo Live* and note the robustness of the contrast transfer function (CTF) determination step for tilted images.

## 2. *Tomo Live* processing workflow

**Figure 3:**
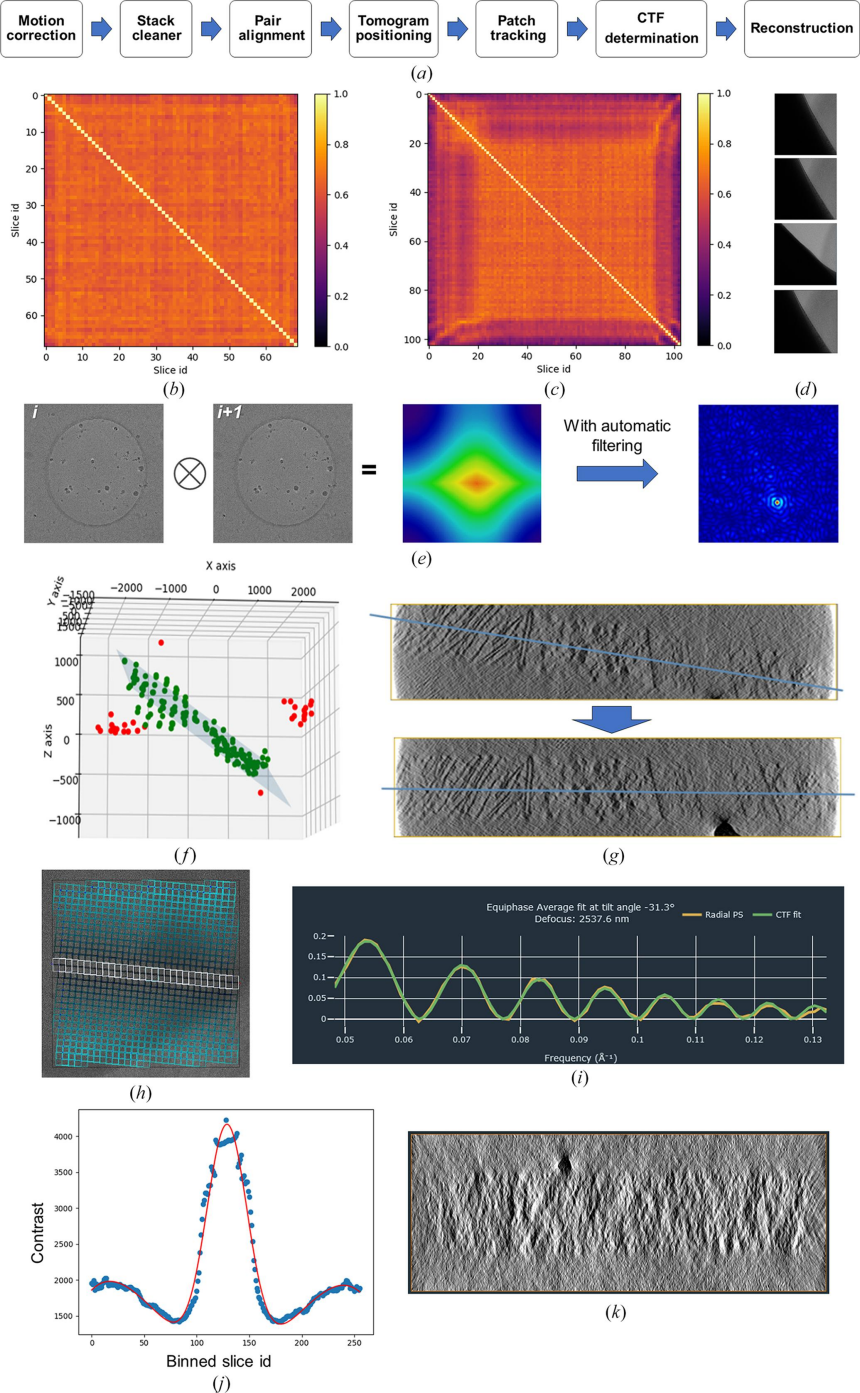
*Tomo Live* workflow. (*a*) Diagram of the processing pipeline. (*b*, *c*, *d*) Stack cleaner showing histogram correlation maps of (*b*) a clean stack without non-usable tilt images and (*c*) a stack with four non-usable tilt images at high tilt angles (*d*). (*e*) Pair-alignment workflow whereby cross-correlation between tilt image *i* and tilt image *i* + 1 is computed; the cross-correlation peak is found to estimate shifts in *X* and *Y*. (*f*, *g*) Tomogram positioning. (*f*) The fitting plan of the 3D positions with outliers shown in red. (*g*) Illustration of tomogram-orientation correction in the *XZ* plane. (*h*, *i*) CTF determination. (*h*) Division of the image into patches parallel to the tilt axis (represented by white patches). (*i*) Representative CTF curve with fitting. (*j*, *k*) 3D reconstruction. (*j*) Contrast function of a binned-down reconstruction. (*k*) Cross section of a reconstruction where the thickness was determined automatically.

The *Tomo Live* processing pipeline is divided into several steps in order to align and reconstruct a tilt series during the acquisition performed by *Tomography* 5. All steps are automatic and no manual interaction is needed. Fig. 3 ▸(*a*) shows a simplified diagram of the processing pipeline as well as examples from key processing steps.

### 2.1. Motion correction

To each MRC/TIFF fraction or EER file (Nakane *et al.*, 2020 ▸) generated by *Tomography* 5, a motion-correction algorithm, based on *MotionCor*2 (Zheng *et al.*, 2017 ▸), is applied to correct for sample movement during the exposure. Global drift correction using 3 × 3 patches and no dose weighting is performed. For EER data, an upsampling of 1 is used. Automatic grouping of frames into fractions and averaging of fractions are performed to reach 1 e^−^ per pixel per fraction.

### 2.2. Stack cleaner

The *Tomo Live *stack cleaner processing step is employed to remove tilt images that are obviously not usable for reconstruction, for example, when grid bars are coming into the field of view (Fig. 3 ▸ *d*). The determination is based on the computation of a correlation matrix using a histogram of gray value correlation. Example correlation matrices are displayed in Figs. 3 ▸(*b*) and 3 ▸(*c*). The mean and standard deviation correlation values are calculated from the matrix. For each tilt image, a check is performed to determine whether the histogram is similar to other tilt images. A tilt image is considered to be usable if the correlation value is greater than a given threshold of mean + 1σ for more than four tilt images. If this criterion is not met, the tilt image is excluded from the tilt series. The algorithm has been tested with both lamellae and SPA samples with and without fiducials. The presence of high-contrast features such as fiducials does not influence the algorithm. Indeed, as long as fiducials are present on all tilt images, these images will not be removed since the histogram will be almost the same. In case of mistracking in some tilt images where a totally different area is imaged, the algorithm will not remove these images if the histogram is almost the same. However, this image will be removed later during the pair-alignment algorithm.

### 2.3. Pair alignment

Pair alignment aims to correct for large shifts occurring along the *X* and *Y* axes. It involves computation of the cross-correlation between tilt images ‘*i*’ and ‘*i* + 1’, with the subsequent identification of the cross-correlation peak (Fig. 3 ▸ *e*). The stack is first reordered by tilt and rotated to correct for the tilt-axis angle. An automated filtering algorithm is applied to optimize the shape of the cross-correlation peak for each pair of tilt images. The shift is determined by measuring the distance between the cross-correlation peak and the center of the image. The pairing starts from the 0° tilt image, where it is always assumed that the zero-sample tilt image is of good enough quality to perform the cross-correlation analysis. Consequently, when no robust cross-correlation peak is found between two successive tilt images, the tilt image with a higher tilt is deemed to be non-usable and the correlation is repeated between tilt images ‘*i*’ and ’*i* + 2’.

### 2.4. Tomogram positioning

The tomogram-positioning step is dedicated to the precise determination of the *X* and *Y* tilt corrections of the tomogram. Subsequently, the *Y* tilt correction is applied to the tilt angles, while the *X* tilt correction is used during the reconstruction step, and the resulting 3D reconstruction is horizontal in the *XZ* and *YZ* cross sections (Fig. 3 ▸ *g*). The algorithm does not require a 3D reconstruction to be performed to determine those angles; only feature tracking is needed. In this case, the fast tracking of features is performed using a dense optical flow algorithm (not cross-correlation as in Section 2.5), where the movement of each pixel is determined. At the end of the tracking, pixel movement within a given patch is averaged. The 3D positions of individual patches are then determined as described in Section 2.6. These positions are then used to determine a plane equation, where the *X* and *Y* angles of the sample are extracted. The plane-fitting algorithm can determine outliers to improve the accuracy of the results (see Fig. 3 ▸ *f*, in which outliers are colored red).

### 2.5. Patch tracking

To determine the alignment parameters that are needed to align each tilt image, fiducial-free tracking utilizing cross-correlation is employed. The initial step involves subdividing the 0° tilt image into 16 patches, each sized at 30% of the input image dimensions. We assume that the 0° tilt image is the horizontal plane of the sample since the tilt angle has been corrected for the sample orientation in the previous step. Subsequently, the patches corresponding to theoretical locations across the tilt series are tracked to generate trajectories. This is performed by cross-correlating neighboring tilt patches, *i.e.* *i* × *i* + 1, *i* + 1 × *i* + 2 *etc*. The *Tomo Live* patch-tracking process operates autonomously. This autonomy is achieved through an algorithm that relies on three parameters, which are described below.

(i) *Automatic patch-movement analysis and replacement*. Given that the sample orientation is established following the tomogram-positioning step and the correction of tilt angles, it becomes feasible to calculate the theoretical shifts of patches between two tilt images,

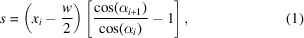

where *x* is the *x* coordinate of a patch, *w* is the image width and *α_i_* is the tilt-angle value for tilt image *i*.

Once the shift between two patches has been determined (called the measured value), this value is considered abnormal if the difference between the measured and theoretical shift value is greater than 2% of the image size. In such instances, the affected patch is replaced to a new position on the image with contrasting features and a new trajectory is created.

To determine the new position, the tilt image is divided into 200 small subpatches. The contrast is then calculated for each of the subpatches, and they are sorted from most to least contrasted. Each patch occupies multiple subpatches, and when a patch is moved to a new position, that position is chosen from the sorted list, starting from the top. The sub­patches occupying the central part of the patch surface, equivalent to 2/3 of the whole patch surface, are labeled as ‘used’. If another patch needs to be moved, the next available unused subpatch is chosen.

(ii) *Automatic determination of filtering parameters for each patch*. Analogous to the pair-alignment step, an automatic bandpass filtering is used to optimize the shape of the cross-correlation peak for each patch. If no cross-correlation peak is found, the patch is repositioned to a new location on the image and a new trajectory is created.

(iii) *Automatic removal of non-usable tilt images*. During tracking, if a specified percentage of patches within a given tilt image necessitate relocation, that particular tilt image is deemed to be ‘non-usable’ and removed. In *Tomo Live*, this threshold is set to 75%.

### 2.6. Determination of the alignment parameters

The trajectories obtained during the patch-tracking step are used to fit the equation that describes the projection images (Frank, 2006 ▸). The variables used in the minimization of the projection model are the rotation, overall scaling factor and image shifts, with the tilt angles of individual tilt images kept constant. The minimization procedure comprises three steps.

(i) *Determination of the tilt-axis angle*. Using the Brent method (Brent, 1973 ▸) to iterate on different values of the tilt-axis angle, minimization of the projection equation is performed with a small number of iterations to determine the initial value of the tilt-axis angle.

(ii) *Determination of the initial alignment matrix without outliers*. For each tilt image, the variables are iteratively determined until convergence is achieved. Convergence is performed on the average residual error between measured and theoretical fiducials. The rotation of each tilt image is initially set using the tilt-axis angle and is subsequently allowed to evolve independently.

(iii) *Removing outlier markers*. Once the first solution of the alignment matrix has been found, a second iteration of the equation is performed, initializing the alignment matrix with the outcome of step (ii). After each iteration, the mean error and standard deviation are calculated, and trajectories are removed if the residual error is greater than three standard deviations. This iterative process is repeated until convergence is reached.

Once the alignment parameters have been determined, the pair-alignment parameters (tilt-axis rotation and shifts) are included and the alignment is performed on the raw stack to minimize interpolation error.

### 2.7. CTF determination

Once all alignment steps have been performed and all non-usable tilt images have been identified, *Tomo Live* determines the CTF parameters from the raw stack where all non-usable tilt images, as identified in the previous steps, have been removed. This avoids having to determine the CTF parameters for tilt images that are not relevant.

In cryo-electron tomography, determining the CTF can be challenging for two main reasons. Firstly, during acquisition the sample is tilted to take images at different tilt angles. For angles that differ from zero, a defocus gradient is present on the sample image in the direction perpendicular to the tilt axis. Moreover, the total dose deposited on the sample is relatively low (up to a maximum of about 140–180 e^−^ Å^−2^ for the series and 2–4 e^−^ Å^−2^ for individual tilt images), resulting in low contrast on the image, making the Fourier transform noisy.

*Tomo Live* can automatically determine, without any user input, the CTF parameters for all tilt images of the tilt series. Firstly, since the sample orientation is determined during the tomogram-positioning step, the algorithm determines the CTF parameters (defocus and astigmatism) on the tilt image where the sample is horizontal to avoid any defocus gradient (*X* tilt is then neglected). For FIB-milled cases, the lamella pre-tilt angle is taken into consideration and the horizontal tilt image is equal to the tilt image with zero sample tilt. The classical 2D CTF fitting described in Rohou & Grigorieff (2015 ▸) is applied to determine the defocus and the astigmatism. For the other tilt images of the tilt series, where a defocus gradient is present, the astigmatism is considered to be constant along the tilt series. The defocus determination for these tilt images is based on the work of Xiong *et al.* (2009 ▸). The principle is to divide the images into rows of patches parallel to the tilt axis (Fig. 3 ▸ *h*). For the same row of patches, the defocus value is assumed to be constant. These patches are averaged to determine the average power spectrum of a row. Then, for each row parallel to the tilt axis, the equiphase average using the astigmatism of the central tilt image is computed. Each computed 1D CTF curve is then rescaled along the frequency domain to have the CTF zeroes at the same frequencies as the CTF curve computed from the row of patches on the tilt axis. The background is modeled with a spline that passes through the zero crossings of the CTF curve and the high-frequency points (Fig. 3 ▸ *i*).

For some data sets where the pixel size is small (<0.5 Å), the low-frequency sampling is low and there are not enough points to describe the CTF oscillations accurately. The image needs to be resampled to improve the signal. To avoid this manual intervention, a simple yet effective automatic algorithm has been developed. This algorithm consists of the following.

(i) Determining the number of points *N*_exp_ needed to describe the first oscillation of the CTF curve.

(ii) This value is then compared with a reference value *N*_ref_ taken for a pixel size of 1.5 nm.

(iii) The resampling value *R* (where *R* is rounded to the nearest half-integer) is determined as

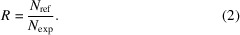




### 2.8. 3D reconstruction

The process of 3D reconstruction relies on the Astra library (van Aarle *et al.*, 2015 ▸), which offers a selection of reconstruction algorithms. As part of its intrinsic design philosophy, *Tomo Live* defaults to utilization of the simultaneous iterative reconstruction technique (SIRT) algorithm (Gilbert, 1972 ▸) due to its notable capacity for noise reduction. Use of SIRT typically results in reconstructions with sufficient contrast for quick quality assessment, which is the overarching objective of *Tomo Live*.

The tomogram thickness is determined automatically based on contrast. Firstly, the aligned stack is binned down to a 256 × 256 pixel image and reconstruction is performed. It is followed by the computation of contrast for each individual *XY* plane. The contrast function is then fitted to determine the tomogram thickness. This thickness value is then provided for the final reconstruction, which is performed on the 4× binned aligned stack.

### 2.9. Integration

The *Tomo Live* pipeline is integrated into the Data Management Platform (DMP) of the microscope (the offload server for images acquired by the detector). Acquisition files, such as MRC/TIF fractions or EER files, are registered in the platform by *Tomography* 5. This registration triggers the *Tomo Live* reconstruction pipeline. At the end of each tilt series, output results, including alignment parameters, aligned tilt and reconstruction stacks, are accessible from the web portal. A dedicated view of the portal utilizing multiple charts allows one to quickly judge the quality of the tomograms. Filtering and export functions will be available to select only relevant data sets for offline processing.

### 2.10. Evaluating data quality and export of *Tomo Live* data

The processing workflow implemented in *Tomo Live* has been optimized for rapid data handling, typically requiring less than 5 min following the acquisition of the last tilt image in a single tilt series. For example, for a typical use case consisting of 41 tilt images recorded at 4k × 4k pixel resolution, SIRT reconstruction (with GPU implementation) takes ∼3 min. Motion correction is performed on the fly as the tilt series is being acquired.

Data files and their associated metadata are then accessible through a web-based interface, facilitating data curation by the user. Metadata plots allow filtering of the data sets based on quality metrics, including Alignment Accuracy, Number of Deleted tilt images (called ‘slices’ in the user interface), Tilt Axis Correction, Defocus, CTF Confidence Range and Astigmatism (Supplementary Fig. S1).

The web-browser interface of *Tomo Live* also allows inspection of the tilt series (motion-corrected and aligned stacks) and the reconstructed tomograms (Supplementary Fig. S2). Following tomogram selection, users can export the raw data as well as data that were generated by *Tomo Live* for further analysis. It is also possible to generate additional files and metadata needed to use *Tomo Live* results in *IMOD* or *RELION* 4.0 (Zivanov *et al.*, 2022 ▸). The additional files generated are as follows.

(i) Alignment parameters in *IMOD* format: *.rawtlt, *.tlt, *.xf, *.tltxf, newst.com, tilt.com.

(ii) The initial CTF parameters in *Ctfplotter* format: *.defocus.

(iii) *RELION*-specific files: the order list per position (*.csv) and the *.star file.

Files in *IMOD* and *Ctfplotter* (Xiong *et al.*, 2009 ▸) formats are directly compatible with *RELION* 4. In cases where the *Tomo Live* data being exported encompass tilt images identified as unusable (Deleted Slices), *Tomo Live* creates a new stack (*clean.mrc) during export, excluding the unusable tilt images. This new stack, as well as other generated files, are then used in *IMOD* or *RELION*. Importing the *Tomo Live* aligned stack to *IMOD* allows the first six steps (from Pre-processing to Tomogram Positioning) to be skipped as the Final Aligned Stack step can be directly used to generate a new aligned stack with a different binning and proceed with a new reconstruction.

## 3. Improvements in the *Tomography* 5 software

The *Tomo Live* processing pipeline complements improvements made in the *Tomography* 5 software for sample navigation and automated data acquisition, all of which focus on ease of use and increased data-collection throughput. Here, we discuss the new sample-navigation features that make adding Batch Positions and multiple exposure areas per single position simpler and faster, increasing throughput. All of the improvements mentioned below have already been released.

### 3.1. Sample navigation

Search Maps are sets of tiles acquired using medium magnification, which allow the mapping of entire grid squares or lamellae. Multiple Search Maps, for example of all lamellae on the grid, can be queued and acquired automatically, similar to Automated Atlas Acquisition. Once acquired, Batch Positions can be added directly onto Search Maps without the need to acquire any additional Search Images. Sample features are accurately centered during automated acquisition using Search Images extracted from the Search Maps.

**Figure 4:**
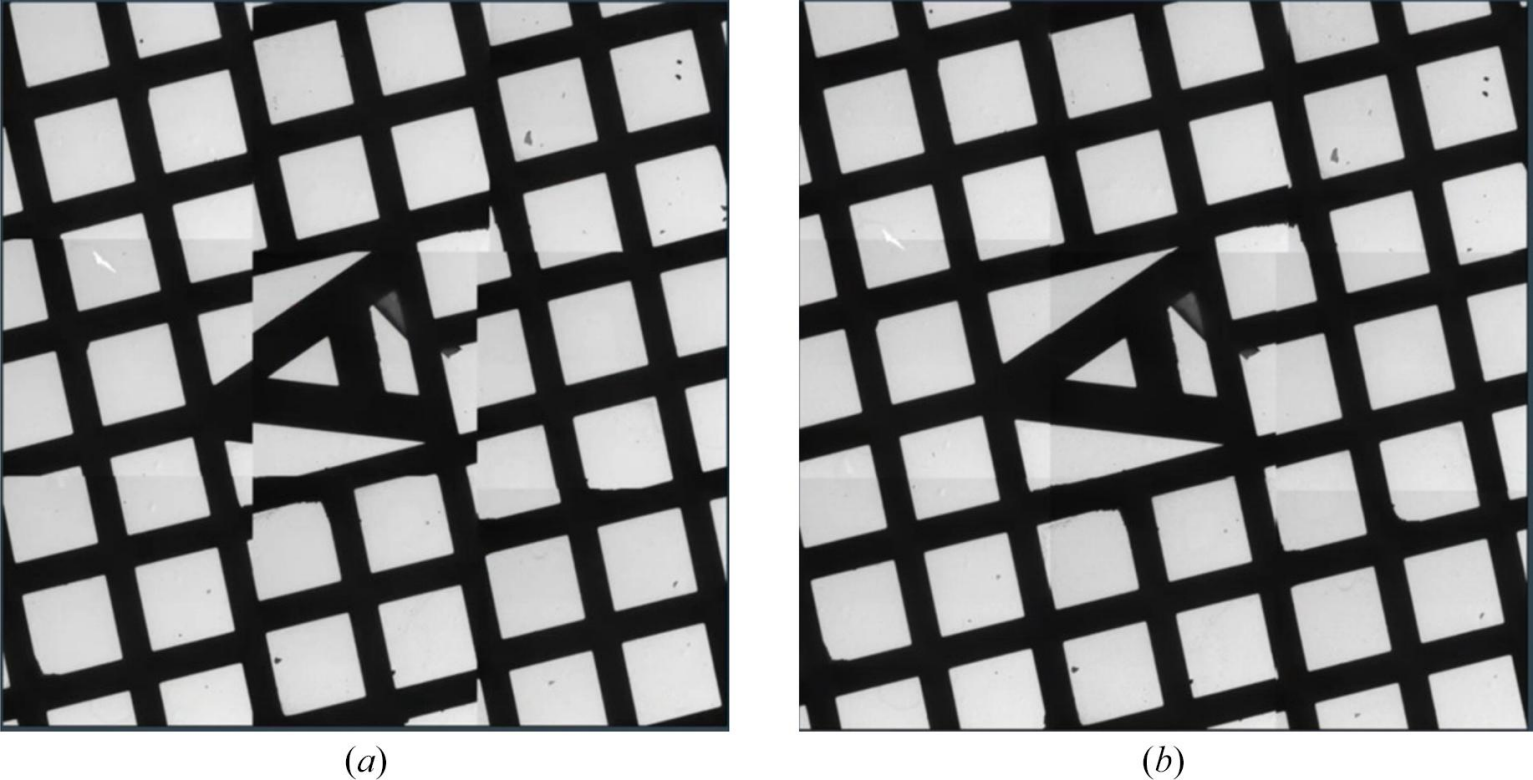
Results of the automated calibration to improve the tile stitching. (*a*) Atlas without the calibration applied, with individual tiles not aligning well with respect to one another. (*b*) Atlas acquired with the calibration applied, showing a significantly improved tile alignment.

While magnification calibration is performed close to focus, imaging further from focus may lead to image rotation and shift. Automated calibration is available to calibrate for settings further from focus and to improve the alignment of individual tiles in Atlases and Search Maps (Fig. 4 ▸). This leads to more accurate navigation, which is especially crucial when acquiring multiple exposure areas during a single Batch Position.

The automated calibration is fast and unattended and measures the difference between the requested stage and/or beam/image shifts and the actual shifts in four directions (±*X*, ±*Y*). The resulting four vectors are combined into a single transformation matrix that is used to correct the navigation on the tile sets. Due to this simple approach, the calibration is fast but has the risk of inaccuracies further away from the center of the tile set.

### 3.2. Multiple exposure areas

**Figure 5:**
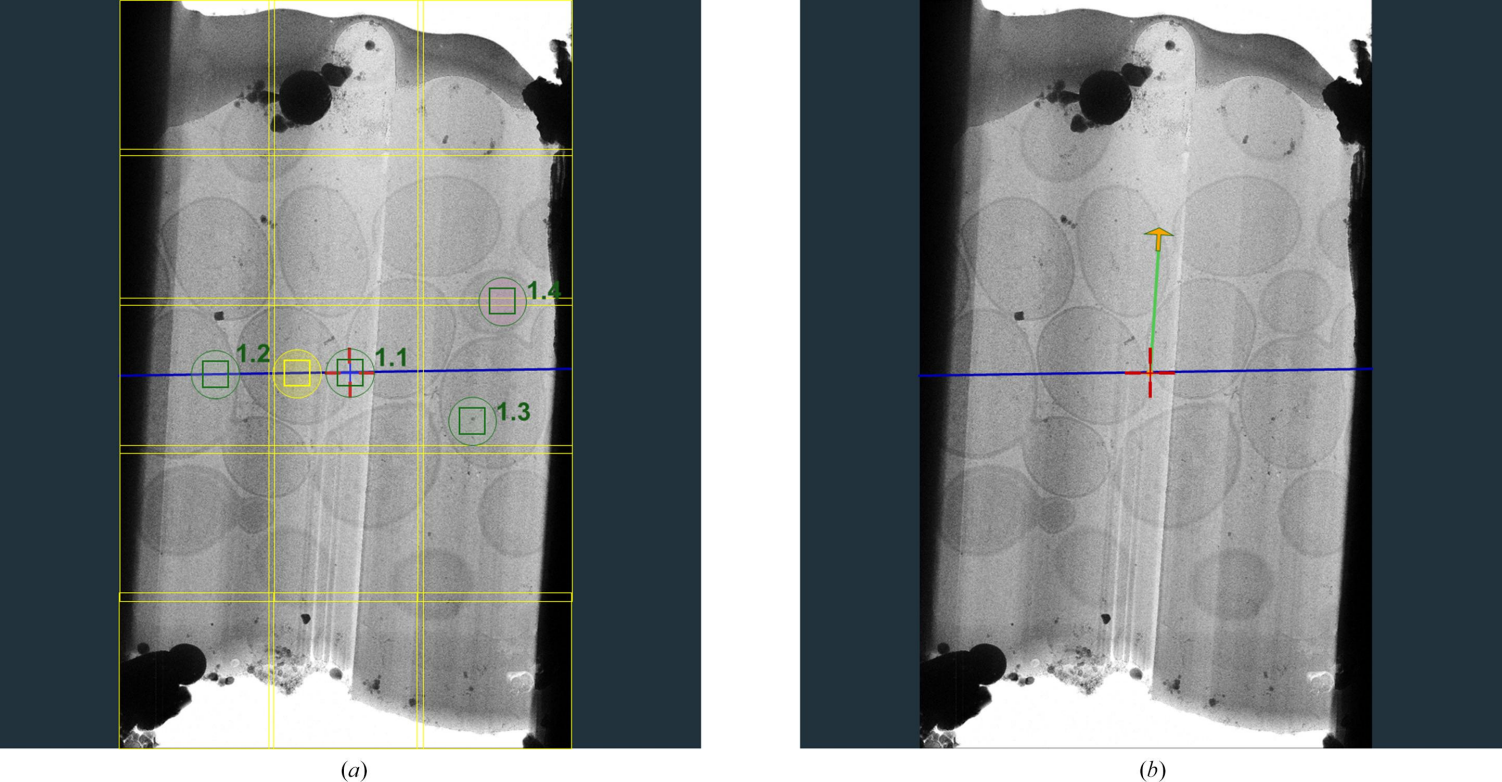
Multiple exposure positioning and definition of lamella orientation. (*a*) Yeast lamella illustrating multiple exposure positioning. 1.1 (central exposure area) and 1.2 denote exposure areas on the stage tilt axis (blue line), whereas 1.3 and 1.4 denote exposure areas positioned away from the tilt axis. (*b*) Defining the lamella orientation (green line) with respect to the stage tilt axis (blue line) to improve tracking.

Utilizing optical image shift allows the collection of multiple tilt series in parallel, thereby saving time and gaining access to sample areas that would otherwise be used up for tracking (Eisenstein *et al.*, 2023 ▸). This is possible in *Tomography* 5 by adding multiple exposure areas for a single Batch Position, where beam/image shift will be used to reach exposure areas away from the central exposure area (Fig. 5 ▸ *a*). A template can be defined to use the same configuration of exposure areas in each Batch Position, as is already possible in the Thermo Scientific *EPU* software. Each exposure area is saved as an individual tilt series and will be processed automatically during the acquisition.

Distances between the individual tilt images of the single exposure area change significantly during the tilt series, and the risk of missing the feature during tracking increases when the distance between the optical axis and the exposure area becomes longer. Therefore, the initial soft limits to position additional (off-axis) exposure areas were set to 6 µm on the tilt axis and 3 µm off the tilt axis from the stage position. It is still possible to add positions further from the stage position; however, above the soft limits the users are warned of the higher possibility of tracking errors, with the color of the exposure area changing to pink.

To demonstrate that good-quality tomograms can be collected off-axis, we acquired tilt series on a keyhole limpet hemocyanin (KLH) sample using 0, 3 and 6 µm image shift. Cryo-electron tomograms from this experiment have been deposited in the EMDB with accession codes EMD-19672 (stage position, 0 µm image shift), EMD-19673 (3 µm image shift) and EMD-19674 (6 µm image shift). A summary of the experimental setup and data-acquisition parameters can be found in the supporting information.

To accurately keep track of multiple exposure areas during the tilt series, the expected movement of each exposure area needs to be calculated using a theoretical movement path of the sample. This becomes more challenging with milled samples, as lamella orientation, pre-tilt and sample deformations play a role in the sample movement during the tilt series (Eisenstein *et al.*, 2023 ▸). To more accurately model the shifts of each individual exposure area throughout the tilt series, the tilt angle and (if necessary) the lamella pre-tilt angle and the orientation of the lamella with respect to the tilt axis need to be specified before the tilt series (Fig. 5 ▸ *b*). All three axes are used to model the shifts in 3D to predict the movement of each feature in three dimensions more accurately. To simplify the calculation, it is assumed that the sample is (relatively) flat and rigid.

In reality, most samples, and especially *in situ* cellular lamellae, show local variations in *Z*-height within the same lamella (Eisenstein *et al.*, 2023 ▸). This can result in large errors in tracking, leading to unprocessable data. To overcome this, the *Z*-height is estimated during the tilt series, the model is updated continuously for each exposure area, and *X* and *Y* shifts per exposure area per tilt are calculated using the more accurate local *Z*-height. By using this approach, additional tracking errors coming from less rigid lamellae are also corrected.

## 4. Case studies

Since the introduction of the software, *Tomo Live* processing and reconstruction have been performed on a variety of samples at the Thermo Fisher Scientific NanoPort facility in Eindhoven. Here, we selected a range of biological specimens, ranging from soluble proteins that are amenable to single-particle analysis to more complex specimens such as pleomorphic virions, whole bacterial cells and cryo-FIB-milled lamella, as representative examples.

### 4.1. Materials and methods

#### 4.1.1. Sample preparation

A frozen aliquot of 6.3 mg ml^−1^ mouse apoferritin in 20 m*M* HEPES pH 7.5, 300 m*M* NaCl, which we received from the Kikkawa laboratory at Tokyo University, was thawed at room temperature. Dithiothreitol (DTT) was added to a final concentration of 1 m*M* prior to grid preparation. Keyhole limpet hemocyanin was purchased from Sigma (catalogue No. H7017) and was resuspended to 10 mg ml^−1^. Wild-type *Magnetospirillum gryphiswaldense*, a magnetotactic bacterium (MTB), was received from the Schüler laboratory at the University of Bayreuth in FSM medium (Heyen & Schüler, 2003 ▸), concentrated by table-top centrifugation and resuspended in growth medium. In all cases, samples were applied to glow-discharged (Quorum GloQube) Quantifoil grids (Quantifoil Micro Tools GmbH) and plunge-frozen into liquid ethane using a Thermo Scientific Vitrobot Mark IV.

Vitrified grids of porcine transmissible gastroenteritis virus (TGEV) and influenza virions were provided by the Martín-Benito Romero laboratory based at CNB–CSIC, Madrid. These samples were prepared as described in previous publications (Cantero *et al.*, 2022 ▸; Arranz *et al.*, 2012 ▸).

*Saccharomyces cerevisiae* (yeast) lamella samples were prepared using a Thermo Scientific Aquilos 2 Cryo-FIB as described previously (Wagner *et al.*, 2020 ▸).

#### 4.1.2. Imaging and reconstruction

More detailed data-collection parameters can be found in Supplementary Table S1. Data acquisition was performed with *Tomography* 5 using either a Thermo Scientific Krios G4 or Glacios 2 cryo-TEM equipped with a Selectris or Selectris X energy filter and a Falcon 4i direct electron detector. Tilt series were acquired using a dose-symmetric tilt scheme (Hagen *et al.*, 2017 ▸) with a 3° step and a group size of 2. *Tomo Live* was used to process all tilt series from raw data to the reconstruction. For the comparison of CTF estimation, motion-corrected tilt series were exported from *Tomo Live* and used for defocus, resolution and astigmatism estimation by *CTFFIND*4 (Rohou & Grigorieff, 2015 ▸). The *CTFFIND*4 search parameters used were as follows: resolution range 15–40 Å, defocus search step 100 nm, defocus range 750–5500 (KLH) or 1500–10000 nm (yeast). For comparison, *Tomo Live* reads the applied defocus value from the microscope and uses this value to initialize the defocus search. The resolution range is tuned automatically.

### 4.2. *Tomo Live* reconstructions

**Figure 6:**
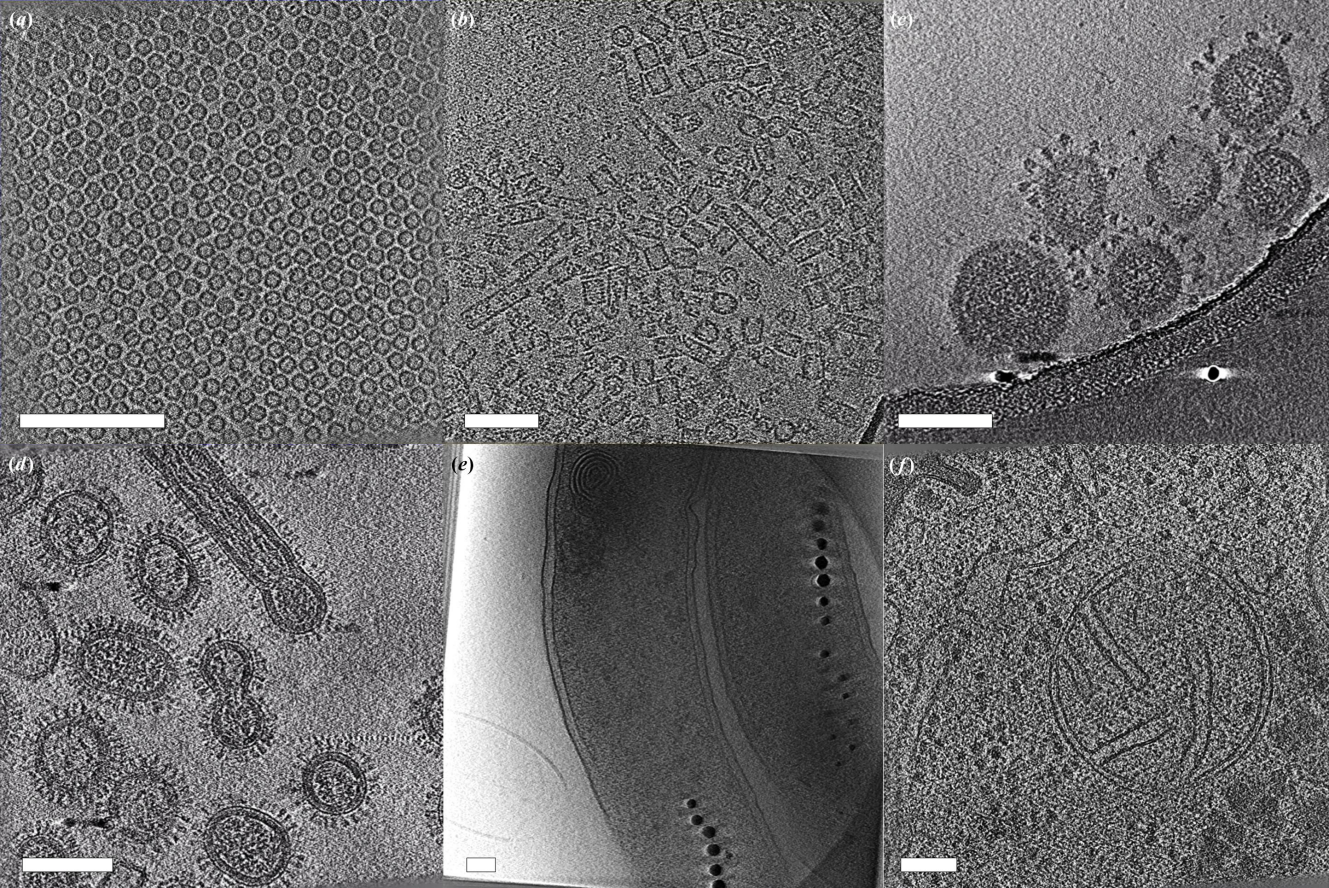
Examples of *Tomo Live* SIRT-reconstructed tomograms. Images from the tomograms of (*a*) apoferritin, (*b*) keyhole limpet hemocyanin, (*c*) TGEV virions, (*d*) influenza virions, (*e*) a magnetotactic bacterium and (*f*) yeast. Scale bars 100 nm.

Six samples in total were chosen as representative examples of *Tomo Live* reconstructions: two SPA samples (apoferritin and KLH), two pleomorphic virus particles (TGEV and influenza), whole magnetotactic bacteria cells and milled yeast cells. Fig. 6 ▸ shows images from the reconstructed tomograms. Cryo-electron tomograms have been deposited in the EMDB with accession codes EMD-19666 (apoferritin), EMD-19667 (KLH), EMD-19668 (TGEV), EMD-19669 (influenza), EMD-19670 (magnetotactic bacterium) and EMD-19671 (yeast). Raw tilt-series data for KLH and yeast have also been deposited in EMPIAR with accession codes EMPIAR-11908 and EMPIAR-11907, respectively.

### 4.3. CTF parameter comparison between *Tomo Live* and *CTFFIND*4

**Figure 7:**
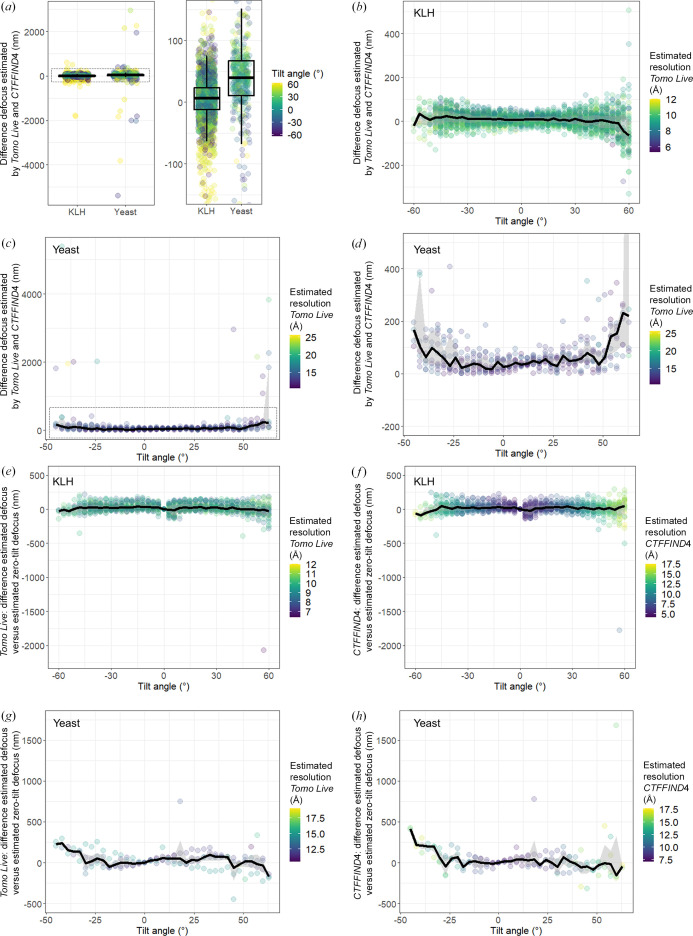
CTF estimation by *Tomo Live* and *CTFFIND*4. (*a*) Estimated defocus by *Tomo Live* minus estimated defocus by *CTFFIND*4 for purified KLH and yeast lamellae (left) for all data points and (right) zoomed-in. Each point denotes one defocus estimate in one tilt image. The color of the points depicts the tilt angle at which the image was recorded. Defocus estimates with estimated resolution accuracy >30 Å for *CTFFIND*4 were excluded. The same results as shown in (*a*) shown per tilt angle for (*b*) KLH and (*c*, *d*) for yeast lamellae: (*c*) all data points and (*d*) zoomed-in. (*e*, *f*) The difference in defocus estimates for higher tilt angles compared with the estimate for the zero-tilt image of the same tilt series for (*e*) *Tomo Live* and (*f*) *CTFFIND*4 for KLH. (*g*, *h*) The difference in defocus estimates for higher tilt angles compared with the estimate for the zero-tilt image of the same tilt series for (*g*) *Tomo Live* and (*h*) *CTFFIND*4 for yeast lamella. Each point denotes one defocus estimate in one tilt image. In all panels, the black line shows the median difference between the two estimates and the gray-shaded area shows the interquartile range.

We compared the robustness of defocus estimation in *Tomo Live* versus *CTFFIND*4 (Rohou & Grigorieff, 2015 ▸; Fig. 7 ▸), which was originally developed for processing untilted SPA images but is nevertheless often used in the pre-processing of tomograms (Bharat & Scheres, 2016 ▸; Scaramuzza & Castaño-Díez, 2021 ▸). For those tilts where *CTFFIND*4 could estimate the defocus reliably (*i.e.* with an estimated resolution accuracy of better than 30 Å), similar defocus estimates were found by both algorithms, especially at lower tilt angles (≤30°), with median absolute differences in estimated defocus of ∼15 and ∼40 nm for tomograms of KLH and yeast lamellae, respectively (Figs. 7 ▸ *a*–7 ▸ *d*). At higher tilt angles (>30°), slightly larger differences were observed between the two algorithms (median absolute differences in estimated defocus of ∼30 and ∼75 nm for tomograms of KLH and yeast lamellae, respectively), and some large outliers (>1 µm difference) for tomograms of yeast lamellae (Figs. 7 ▸ *e*–7 ▸ *h*). While for *Tomo Live* the estimated defocus shows little deviation from the zero-tilt image for the higher tilt images in the same tilt series, which is what one would expect given that the applied defocus should have been constant throughout the recording of the whole tomogram, larger deviation outliers were observed for *CTFFIND*4 for tomograms of yeast lamellae. This suggests that *Tomo Live* estimates defocus for tilted images more accurately in these cases, while both methods perform similarly at low tilt angles. One possible explanation is that unlike *Tomo Live*, *CTFFIND*4 does not account for the difference in defocus across images from tilted specimens, which causes blurring of the Thon rings.

For SPA applications on tilted data or (high-resolution) subtomogram averaging, such errors in CTF estimation would be corrected in subsequent CTF refinement. However, reliable CTF estimates are still important when corrected tomograms are immediately used in other applications, such as morphological studies.

**Figure 8:**
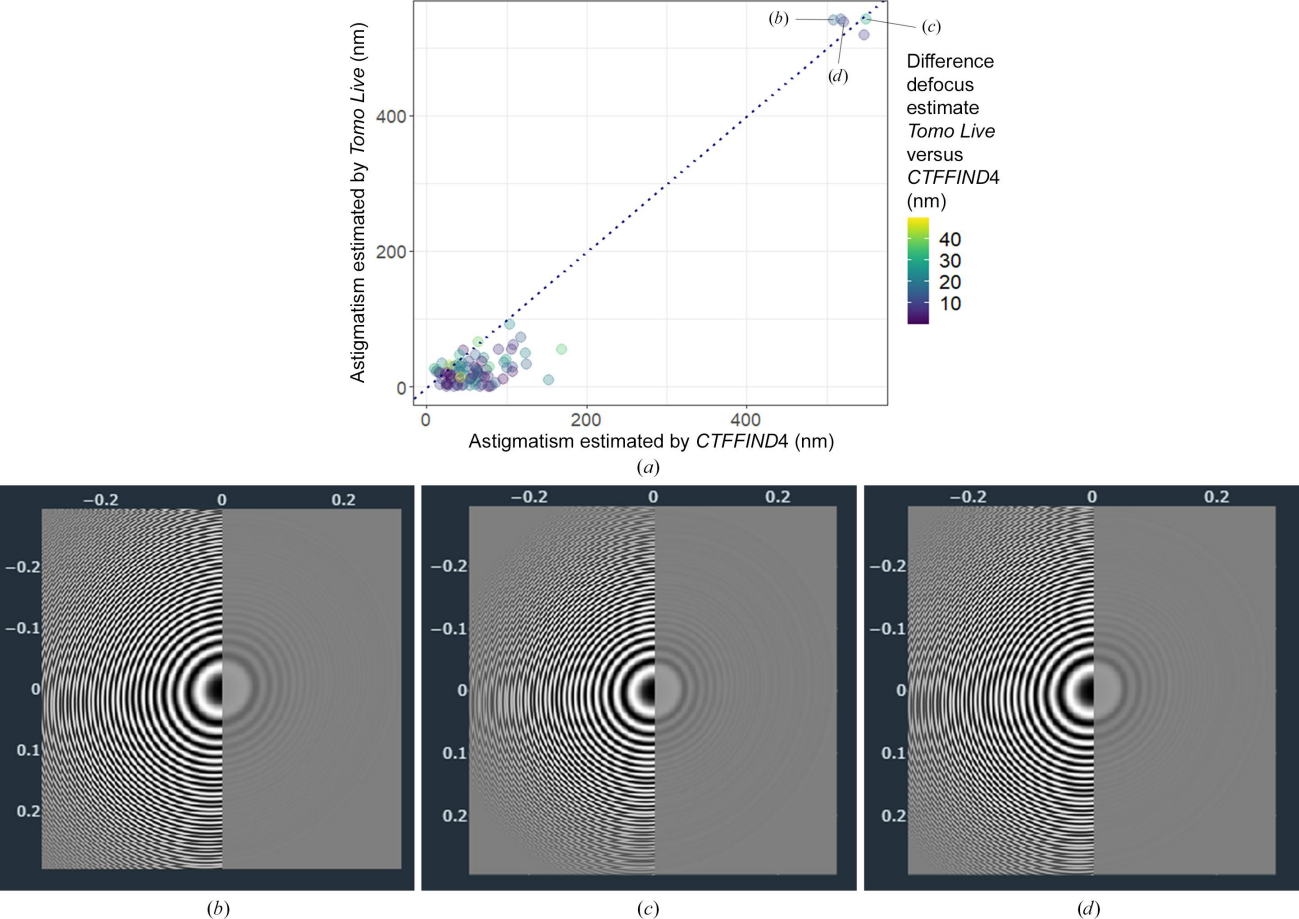
(*a*) Estimated astigmatism by *Tomo Live* plotted against estimated astigmatism by *CTFFIND*4. Each data point corresponds to one tilt series. Some tomograms were purposefully collected with strong astigmatism, as shown in (*b*), (*c*) and (*d*), which show power spectra displayed in the *Tomo Live* web interface.

The astigmatism estimated by *Tomo Live* was similar to that estimated by *CTFFIND*4, with a median absolute difference of 30 nm (Fig. 8 ▸). For tomograms purposefully collected with strong astigmatism (as shown by the power spectra), both algorithms correctly identified the high astigmatism.

## 5. Discussion

Cryo-electron tomography has emerged as a powerful tool in structural biology, offering unparalleled insights into the nanoscale architecture of biological specimens. Nevertheless, the efficiency and user-friendliness of the cryo-ET data-acquisition and processing workflow still trail behind those of SPA. Recent strides in SPA have benefited significantly from the incorporation of real-time data processing, which facilitates efficient sample screening, early recognition of sub­optimal data and the dynamic optimization of data-acquisition parameters.

In response to the demand for similar advancements in cryo-ET, we developed *Tomo Live*, an on-the-fly tomography data-processing software which runs directly on the microscope hardware. *Tomo Live* autonomously performs motion correction, tilt-series alignment and 3D reconstruction in real time, allowing users to make on-the-fly assessments of data quality, identify data sets unsuitable for further analysis and export curated data sets for processing through a user-friendly web-based interface.

Moreover, *Tomo Live* represents a substantial leap forward by bridging the gap between data acquisition and processing. It provides a platform for future automation, enabling potential feedback loops and increasing the user-friendliness and efficiency of the entire cryo-ET workflow. In this context, *Tomo Live* complements existing developments in the cryo-ET field, addressing a critical need for data curation and automated data processing. It aligns with a broader trend in the field that seeks to accelerate progress by simplifying the workflows, making cryo-ET more accessible to researchers of diverse expertise and contributing to the ongoing acceleration of the cryo-ET field. Future developments in *Tomo Live* will be focused on improving automation (such as automatically determining the pre-tilt angle based on defocus gradient), adding additional quality metrics such as an ice-quality estimation, extending the list of export formats and introducing options for reprocessing tomograms with different reconstruction parameters.

*Tomography* 5 and *Tomo Live* are proprietary software and are available exclusively on Thermo Fisher TEM instruments. We invite and encourage the submission of general feedback, support requests and any other comments or questions related to the *Tomography* 5 and/or *Tomo Live* software through our designated email address: Tomo5@thermofisher.com.

## Supplementary Material

EMDB reference: apoferritin, EMD-19666

EMDB reference: keyhole limpet hemocyanin, EMD-19667

EMDB reference: EMD-19672

EMDB reference: EMD-19673

EMDB reference: EMD-19674

EMDB reference: porcine transmissible gastroenteritis virus, EMD-19668

EMDB reference: influenza virions, EMD-19669

EMDB reference: magnetotactic bacteria, EMD-19670

EMDB reference: yeast lamellae, EMD-19671

Supplementary Figures and Table. DOI: 10.1107/S2059798324001840/qv5004sup1.pdf

## References

[bb1] Aarle, W. van, Palenstijn, W. J., De Beenhouwer, J., Altantzis, T., Bals, S., Batenburg, K. J. & Sijbers, J. (2015). *Ultramicroscopy*, **157**, 35–47. 10.1016/j.ultramic.2015.05.002 26057688

[bb2] Arranz, R., Coloma, R., Chichón, F. J., Conesa, J. J., Carrascosa, J. L., Valpuesta, J. M., Ortín, J. & Martín-Benito, J. (2012). *Science*, **338**, 1634–1637. 10.1126/science.1228172 23180776

[bb3] Balyschew, N., Yushkevich, A., Mikirtumov, V., Sanchez, R. M., Sprink, T. & Kudryashev, M. (2023). *Nat Commun*, **14**, 6543. 10.1038/s41467-023-42085-w PMC10582028 37848413

[bb4] Bepler, T., Borst, A. J., Bouvette, J., Cannone, G., Chen, S., Cheng, A., Cheng, A., Fan, Q., Grollios, F., Gupta, H., Gupta, M., Humphreys, T., Kim, P. T., Kuang, H., Li, Y., Noble, A. J., Punjani, A., Rice, W. J., Oscar, S., Sorzano, C., Stagg, S. M., Strauss, J., Yu, L., Carragher, B. & Potter, C. S. (2022). *J. Struct. Biol.* **214**, 107913. 10.1016/j.jsb.2022.107913 36341954

[bb5] Berger, C., Dumoux, M., Glen, T., Yee, N. B., Mitchels, J. M., Patáková, Z., Darrow, M. C., Naismith, J. H. & Grange, M. (2023). *Nat. Commun.* **14**, 629. 10.1038/s41467-023-36372-9 PMC9902539 36746945

[bb6] Bharat, T. A. M. & Scheres, S. H. W. (2016). *Nat. Protoc.* **11**, 2054–2065. 10.1038/nprot.2016.124 PMC5215819 27685097

[bb7] Bouvette, J., Huang, Q., Riccio, A. A., Copeland, W. C., Bartesaghi, A. & Borgnia, M. J. (2022). *eLife*, **11**, e80047. 10.7554/eLife.80047 PMC9398423 35997703

[bb8] Brent, R. P. (1973). *Algorithms for Minimization Without Derivatives*, pp. 61–80. Englewood Cliffs: Prentice Hall.

[bb9] Caesar, J., Reboul, C. F., Machello, C., Kiesewetter, S., Tang, M. L., Deme, J. C., Johnson, S., Elmlund, D., Lea, S. M. & Elmlund, H. (2020). *J. Struct. Biol. X*, **4**, 100040. 10.1016/j.yjsbx.2020.100040 PMC7695977 33294840

[bb10] Cantero, M., Carlero, D., Chichón, F. J., Martín-Benito, J. & De Pablo, P. J. (2022). *Cells*, **11**, 1759. 10.3390/cells11111759 PMC9179875 35681454

[bb11] Cheng, A., Kim, P. T., Kuang, H., Mendez, J. H., Chua, E. Y. D., Maruthi, K., Wei, H., Sawh, A., Aragon, M. F., Serbynovskyi, V., Neselu, K., Eng, E. T., Potter, C. S., Carragher, B., Bepler, T. & Noble, A. J. (2023). *IUCrJ*, **10**, 77–89. 10.1107/S2052252522010624 PMC9812217 36598504

[bb12] Eisenstein, F., Yanagisawa, H., Kashihara, H., Kikkawa, M., Tsukita, S. & Danev, R. (2023). *Nat. Methods*, **20**, 131–138. 10.1038/s41592-022-01690-1 36456783

[bb13] Frank, J. (2006). Editor. *Electron Tomography: Methods for Three-Dimensional Visualization of Structures in the Cell*, 2nd ed. New York: Springer.

[bb14] Gilbert, P. (1972). *J. Theor. Biol.* **36**, 105–117. 10.1016/0022-5193(72)90180-4 5070894

[bb15] Hagen, W. J. H., Wan, W. & Briggs, J. A. G. (2017). *J. Struct. Biol.* **197**, 191–198. 10.1016/j.jsb.2016.06.007 PMC5287356 27313000

[bb16] Heyen, U. & Schüler, D. (2003). *Appl. Microbiol. Biotechnol.* **61**, 536–544. 10.1007/s00253-002-1219-x 12764570

[bb17] Hylton, R. K. & Swulius, M. T. (2021). *iScience*, **24**, 102959. 10.1016/j.isci.2021.102959 PMC8383006 34466785

[bb18] Khavnekar, S., Erdmann, P. & Wan, W. (2023). *Microsc. Microanal.* **29**, 1020.

[bb19] Kimanius, D., Dong, L., Sharov, G., Nakane, T. & Scheres, S. H. W. (2021). *Biochem. J.* **478**, 4169–4185. 10.1042/BCJ20210708 PMC8786306 34783343

[bb20] Liu, H.-F., Zhou, Y., Huang, Q., Piland, J., Jin, W., Mandel, J., Du, X., Martin, J. & Bartesaghi, A. (2023). *Nat. Methods*, **20**, 1909–1919. 10.1038/s41592-023-02045-0 PMC10703682 37884796

[bb21] Mastronarde, D. N. & Held, S. R. (2017). *J. Struct. Biol.* **197**, 102–113. 10.1016/j.jsb.2016.07.011 PMC5247408 27444392

[bb22] Nakane, T., Kotecha, A., Sente, A., McMullan, G., Masiulis, S., Brown, P. M. G. E., Grigoras, I. T., Malinauskaite, L., Malinauskas, T., Miehling, J., Uchański, T., Yu, L., Karia, D., Pechnikova, E. V., de Jong, E., Keizer, J., Bischoff, M., McCormack, J., Tiemeijer, P., Hardwick, S. W., Chirgadze, D. Y., Murshudov, G., Aricescu, A. R. & Scheres, S. H. W. (2020). *Nature*, **587**, 152–156. 10.1038/s41586-020-2829-0 PMC7611073 33087931

[bb23] Punjani, A., Rubinstein, J. L., Fleet, D. J. & Brubaker, M. A. (2017). *Nat. Methods*, **14**, 290–296. 10.1038/nmeth.4169 28165473

[bb24] Pyle, E. & Zanetti, G. (2021). *Biochem. J.* **478**, 1827–1845. 10.1042/BCJ20200715 PMC8133831 34003255

[bb25] Rigort, A., Bäuerlein, F. J. B., Villa, E., Eibauer, M., Laugks, T., Baumeister, W. & Plitzko, J. M. (2012). *Proc. Natl Acad. Sci. USA*, **109**, 4449–4454. 10.1073/pnas.1201333109 PMC3311327 22392984

[bb26] Rohou, A. & Grigorieff, N. (2015). *J. Struct. Biol.* **192**, 216–221. 10.1016/j.jsb.2015.08.008 PMC6760662 26278980

[bb27] Scaramuzza, S. & Castaño-Díez, D. (2021). *PLoS Biol.* **19**, e3001318. 10.1371/journal.pbio.3001318 PMC8389376 34437529

[bb28] Schenk, A. D., Cavadini, S., Thomä, N. H. & Genoud, C. (2020). *J. Chem. Inf. Model.* **60**, 2561–2569. 10.1021/acs.jcim.9b01102 32233514

[bb29] Tacke, S., Erdmann, P., Wang, Z., Klumpe, S., Grange, M., Plitzko, J. & Raunser, S. (2021). *J. Struct. Biol.* **213**, 107743. 10.1016/j.jsb.2021.107743 33971286

[bb31] Tang, G., Peng, L., Baldwin, P. R., Mann, D. S., Jiang, W., Rees, I. & Ludtke, S. J. (2007). *J. Struct. Biol.* **157**, 38–46. 10.1016/j.jsb.2006.05.009 16859925

[bb32] Tegunov, D. & Cramer, P. (2019). *Nat. Methods*, **16**, 1146–1152. 10.1038/s41592-019-0580-y PMC6858868 31591575

[bb33] Turk, M. & Baumeister, W. (2020). *FEBS Lett.* **594**, 3243–3261. 10.1002/1873-3468.13948 33020915

[bb34] Wagner, F. R., Watanabe, R., Schampers, R., Singh, D., Persoon, H., Schaffer, M., Fruhstorfer, P., Plitzko, J. & Villa, E. (2020). *Nat. Protoc.* **15**, 2041–2070. 10.1038/s41596-020-0320-x PMC8053421 32405053

[bb35] Xiong, Q., Morphew, M. K., Schwartz, C. L., Hoenger, A. H. & Mastronarde, D. N. (2009). *J. Struct. Biol.* **168**, 378–387. 10.1016/j.jsb.2009.08.016 PMC2784817 19732834

[bb36] Zheng, S., Wolff, G., Greenan, G., Chen, Z., Faas, F. G. A., Bárcena, M., Koster, A. J., Cheng, Y. & Agard, D. A. (2022). *J. Struct. Biol. X*, **6**, 100068. 10.1016/j.yjsbx.2022.100068 PMC9117686 35601683

[bb37] Zheng, S. Q., Palovcak, E., Armache, J.-P., Verba, K. A., Cheng, Y. & Agard, D. A. (2017). *Nat. Methods*, **14**, 331–332. 10.1038/nmeth.4193 PMC5494038 28250466

[bb38] Zivanov, J., Otón, J., Ke, Z., von Kügelgen, A., Pyle, E., Qu, K., Morado, D., Castaño-Díez, D., Zanetti, G., Bharat, T. A., Briggs, J. A. & Scheres, S. H. W. (2022). *eLife*, **11**, e83724. 10.7554/eLife.83724 PMC9815803 36468689

