# Supplementary material for: Tomo Live: an on-the-fly reconstruction pipeline to judge data quality for cryo-electron tomography workflows
Source: Acta Crystallogr D Struct Biol. 2024 Mar 21;80(Pt 4):247–58. doi: 10.1107/S2059798324001840 (PMC10994173; doi:10.1107/S2059798324001840)
Supplement: Supplementary file 1 [file d-80-00247-sup1.pdf]

STRUCTURAL BIOLOGY

Volume 80 (2024)

Supporting information for article:

Tomo Live: an on-the-fly reconstruction pipeline to judge data quality for cryo-electron tomography workflows

Maxime Comet, Patricia M. Dijkman, Reint Boer Iwema, Tilman Franke, Simonas Masiulis, Ruud Schampers, Oliver Raschdorf, Fanis Grollios, Edward E. Pryor and Ieva Drulyte

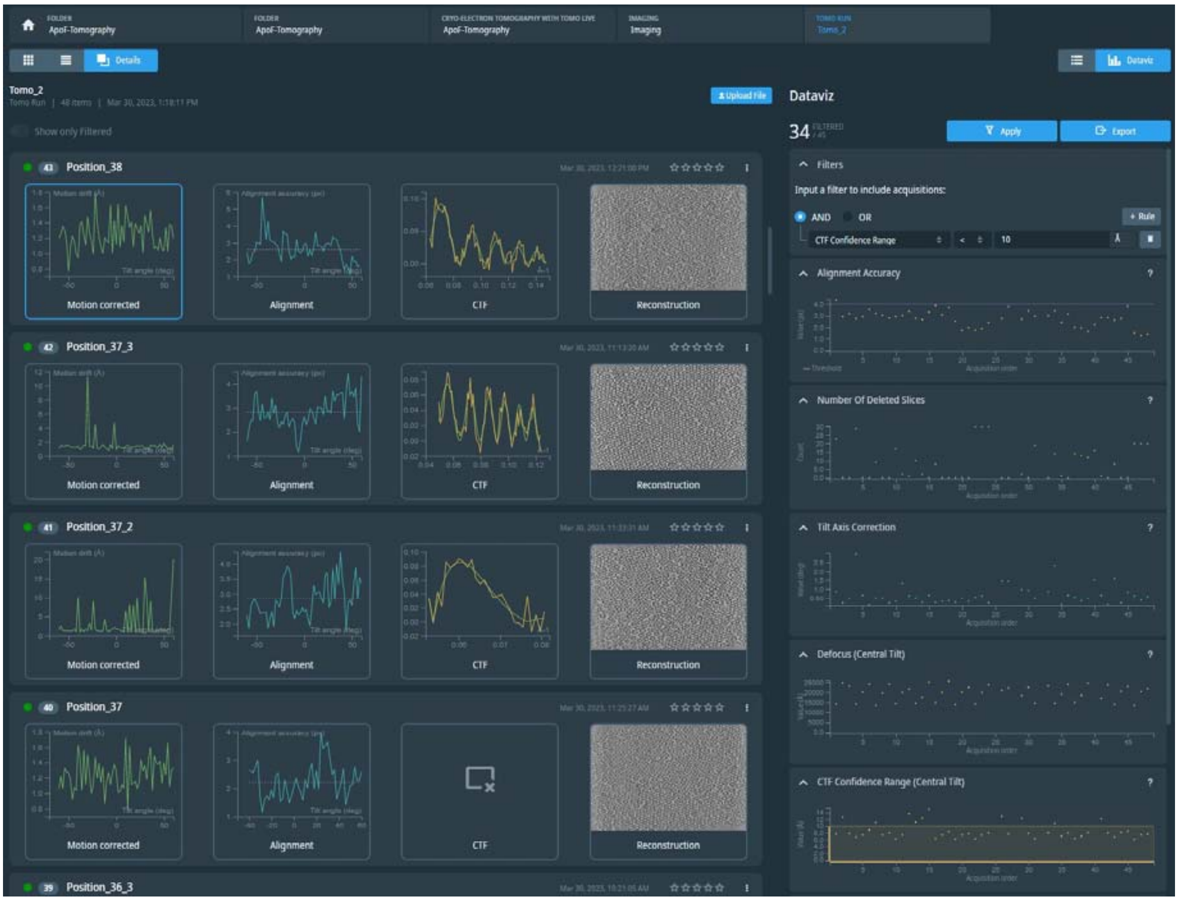

Figure S1 Data curation in Tomo Live.

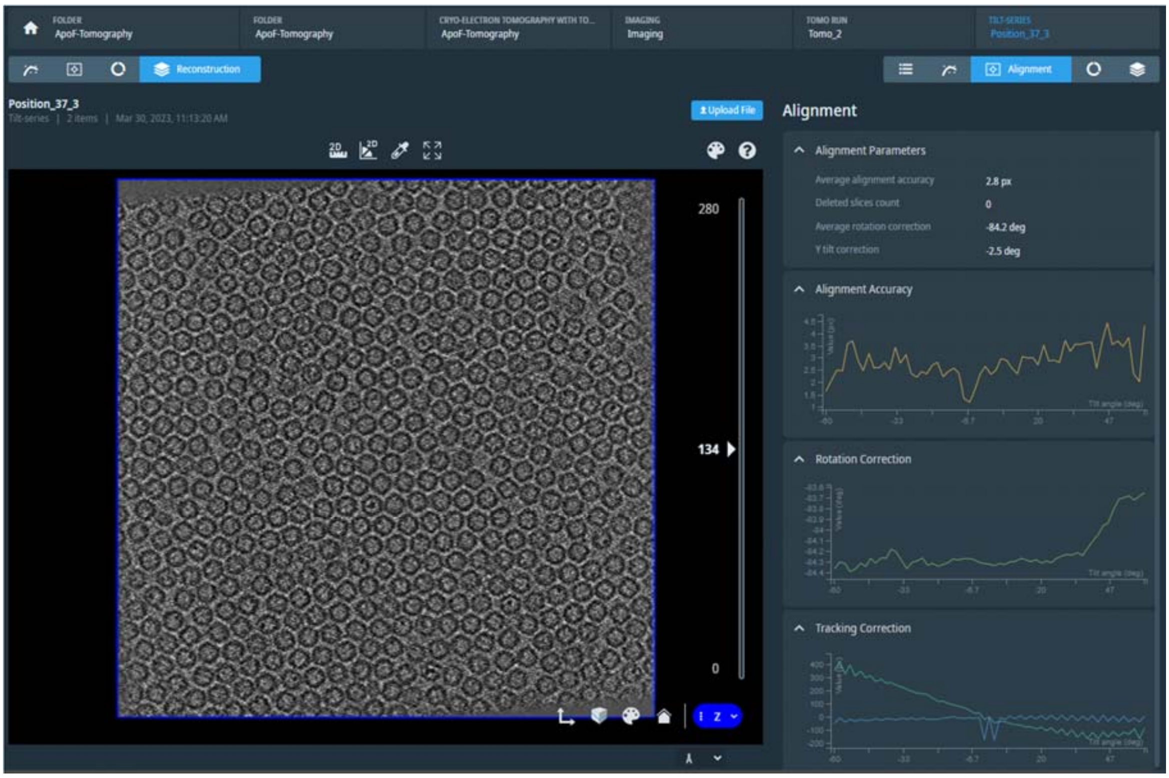

Figure S2 Reconstruction inspection step.

Table S1 Sample details and data collection parameters.

Sample	Apoferritin	KLH	TGEV	Influenza	MTB	Yeast
Accelerated voltage (kV)	300	300	200	200	300	300
Camera	Falcon 4i	Falcon 4i	Falcon 4i	Falcon 4i	Falcon 4i	Falcon 4i
Energy filter	Selectris X	Selectris X	Selectris	Selectris	Selectris X	Selectris X
Slit (eV)	10	10	10	10	10	10
Nominal magnification	165,000x	81,000x	105,000x	105,000x	33,000x	64,000x
Nominal pixel size (Å/pix)	0.75	1.5	1.18	1.22	3.76	1.97
Total dose (e^-/Å^2)	120	102.5	150	150	150	140
Tilt scheme	Dose symmetric	Dose symmetric	Dose symmetric	Dose symmetric	Dose symmetric	Dose symmetric (9° start angle)
Tilt span	120°	120°	96°	108°	120°	108°
Tilt step	3°	3°	3°	3°	3°	3°
Accession codes	EMD-19666	EMD-19667<br>EMPIAR-11908	EMD-19668	EMD-19669	EMD-19670	EMD-19671<br>EMPIAR-11907
